# ZDHHC5 may regulate the function of NKT cells and the immune response in Meniere’s disease

**DOI:** 10.1007/s00335-026-10264-x

**Published:** 2026-08-13

**Authors:** Ruilong Li, Keguang Chen

**Affiliations:** 1 Department of Otorhinolaryngology, Zhongshan Hospital (Xiamen), Fudan University, Xiamen, 361000 China; 2 Department of Otorhinolaryngology-Head and Neck Surgery, Zhongshan Hospital, Fudan University, Shanghai, 200032 China; 3 Xiamen Clinical Research Center for Cancer Therapy, Xiamen, 361000 China

## Abstract

Meniere’s disease (MD) is a heterogeneous, rare inner ear disorder characterized by recurrent vertigo, fluctuating sensorineural hearing loss, tinnitus, and aural fullness. Its molecular mechanisms remain unclear due to significant clinical and immunological heterogeneity. In this study, we integrated multi‑omics genetic data to systematically screen for palmitoylation regulatory genes associated with MD risk. We first intersected 31 core palmitoylation genes with blood cis‑eQTL datasets to identify candidate expression‑related genes, then performed two‑sample Mendelian randomization (MR) to examine their genetic correlation with MD. Summary‑data‑based MR (SMR) using two independent peripheral blood eQTL cohorts (eQTLGen and GTEx) was further applied to validate robust candidate genes. Two‑sample inverse‑variance weighted (IVW) MR revealed nominally significant associations between PPT2, ZDHHC18, ZDHHC5 and MD risk. Independent SMR validation confirmed that peripheral ZDHHC5 expression was significantly correlated with MD (eQTLGen: PSMR = 0.023; GTEx: PSMR = 0.006), and a non‑significant HEIDI test ruled out strong linkage disequilibrium confounding. We further explored potential immune mediators using a two‑step MR screening framework covering 731 peripheral immune cell phenotypes. After Benjamini–Hochberg false discovery rate (FDR) correction for all immune traits to control for multiple testing, no immune cell phenotype retained an FDR < 0.05 association with MD; therefore, formal mediation analysis was not conducted. In summary, this MR‑based genetic analysis identifies ZDHHC5 as a gene associated with MD susceptibility. The potential immune regulatory axis involving NKT cells remains speculative and requires validation through large‑scale stratified immune cohort data and functional cellular/animal experiments. No definitive causal or therapeutic conclusions can be drawn from the present genetic statistical evidence alone.

## Introduction

Meniere’s disease (MD) is not a single homogeneous clinical entity but rather an umbrella diagnosis encompassing a group of rare inner ear disorders. Its core clinical syndrome consists of episodic vertigo accompanied by fluctuating sensorineural hearing loss, tinnitus, and aural fullness (Harcourt et al. 2014, Nakashima et al. 2016). Vertigo attacks typically last from 20 min to several hours, and progressive auditory and vestibular deterioration significantly impairs patients’quality of life (Mohseni-Dargah et al. 2023). The histopathological hallmark of MD is endolymphatic hydrops; however, the molecular mechanisms driving its formation are highly heterogeneous, with distinct pathogenic pathways operating in different patient subgroups (Yamamoto et al. 2010, Lopez-Escamez and Perez-Carpena 2024).

Traditional etiological hypotheses have focused on an imbalance between endolymph production and absorption, vascular dysfunction, genetic susceptibility, and immune-mediated mechanisms (Yuan et al. 2025, Chiarella et al. 2015, Greco et al. 2012, Frejo et al. 2017). In recent years, accumulating evidence from multi-omics and clinical immunological studies has strongly supported a central role of immune dysregulation in the pathogenesis of MD. Independent cohort studies in European and Chinese populations have identified distinct immunophenotypes in sporadic MD: an allergic inflammatory subtype mediated by T helper 2 (Th2) cells, an autoinflammatory subtype characterized by elevated interleukin-1β (IL-1β), and a subtype with concomitant systemic autoimmune disease. These findings highlight the marked immunological heterogeneity of the disease (Lopez-Escamez and Cruz-Granados 2025, Frejo et al. 2025). Further studies have confirmed the involvement of multiple immune cell subsets in the development and progression of MD, including CD4⁺T-cell subset imbalance, natural killer (NK) cell dysfunction, macrophage secretion of pro-inflammatory cytokines, and activation of the NLRP3 inflammasome pathway (Flook et al. 2023, Flook et al. 2024, Jiang et al. 2025, Cruz-Granados et al. 2024, Peng et al. 2026, Liu et al. 2026, Li et al. 2026, Zhang et al. 2024). Notably, NKT cells—an innate-like T-cell subset that bridges innate and adaptive immunity—remain poorly investigated in MD, and the upstream regulatory mechanisms underlying these immune abnormalities are still largely unknown.

Over the past decade, protein post-translational modifications (PTMs) have increasingly emerged as critical regulatory nodes in immune cell function and inflammatory signaling. Protein palmitoylation is a reversible PTM mediated by the ZDHHC family of protein acyltransferases (PATs), in which a palmitoyl group (C16:0) is covalently attached to cysteine residues of substrate proteins, thereby dynamically regulating protein trafficking, membrane localization, stability, and signal transduction (Salaun et al. 2010, Fuller et al. 2023). The ZDHHC family comprises 23 acyltransferase members, which, together with depalmitoylating enzymes such as the palmitoyl-protein thioesterase (PPT) family, form a complete“writer–eraser”regulatory cycle that governs global cellular palmitoylation levels (Liao et al. 2024, Chen et al. 2024).

A growing body of evidence indicates that protein palmitoylation exerts broad immunoregulatory functions by modulating T cell receptor (TCR) signaling, the JAK-STAT cytokine pathway, and NLRP3 inflammasome activation, thereby influencing immune cell development, differentiation, and effector functions (Shi et al. 2022, Zheng et al. 2023, Cao et al. 2025). Dysregulated palmitoylation has been implicated in the pathogenesis of various immune-related and inflammatory diseases. Among the PAT family, ZDHHC5—one of the most functionally well-characterized members—has been shown to regulate innate immune responses, pyroptosis, and the progression of inflammatory diseases (Zheng et al. 2023, Xiu et al. 2025). Based on the core feature of immune dysregulation in MD, we hypothesize that palmitoylation-regulating genes may contribute to genetic susceptibility to MD.

Observational studies are inherently limited by confounding bias and reverse causation, which precludes robust causal inference (Sekula et al. 2016). Mendelian randomization (MR) is a well-established epidemiological approach that uses genetic variants as instrumental variables (IVs) to infer causal relationships between exposures and outcomes, thereby mitigating the confounding bias and reverse causation commonly encountered in conventional observational studies (Smith and Ebrahim 2003). As an extension of MR, summary-data-based Mendelian randomization (SMR) integrates genome-wide association study (GWAS) summary statistics with expression quantitative trait loci (eQTL) data, enabling efficient screening of causal genes for complex diseases and identification of potential therapeutic targets (Zhu et al. 2016).

Given that immune dysregulation is a core feature of sporadic MD and that palmitoylation broadly modulates immune signaling cascades, we hypothesized that palmitoylation-related genetic variants may regulate peripheral immune cell function and contribute to the genetic susceptibility of sporadic MD. Notably, previous genetic studies on familial MD have identified inner-ear structural genes such as OTOG, TECTA, and MYO7A, which belong to pathways entirely distinct from the immune-regulatory gene ZDHHC5 identified in this study. Moreover, there is currently no direct functional experimental evidence in the literature linking the ZDHHC5 palmitoylation pathway to NKT cell function. In this study, we employed two-sample Mendelian randomization (MR) combined with independent-cohort SMR validation to systematically screen for genetic associations between palmitoylation-related genes and MD. We further constructed a two-step immune mediation screening framework to explore potential immune pathways. However, after full-dimensional FDR multiple-testing correction, no reliable immune-associated targets were identified; thus, the relevant immune mechanisms are discussed only as hypotheses.

## Materials and methods

### Data acquisition and rationale

#### Acquisition of eQTL genes

All gene expression quantitative trait locus (eQTL) data used in this study were obtained from the eQTLGen database (https://www.eqtlgen.org/cis-eqtls.html) (Westra et al. 2013). The dataset consisted of cis-eQTLs derived exclusively from blood samples. To ensure the reliability and validity of the instrumental variables, a genome-wide significance threshold (*P* < 5 × 10⁻⁸) was applied to filter single nucleotide polymorphisms (SNPs), with linkage disequilibrium clumping parameters set at R^2^ < 0.1 and a clumping window of 10,000 kb. This approach minimized redundancy and confounding effects due to linkage disequilibrium, thereby selecting the most representative SNPs. To mitigate bias from weak instrumental variables, the F-statistic was calculated for each SNP, and only those with an F-statistic > 10 were retained as robust instruments (Chen et al. 2024). The F-statistic was computed as follows: F = R^2^ × (*N* − 2)/(1 − R^2^), where R² = 2 × (Beta)^2^ × EAF × (1 − EAF)/[2 × (Beta)^2^ × EAF × (1 − EAF) + 2 × (SE)^2^ × N × EAF × (1 − EAF)] (Yang et al. 2023). Here, N represents the sample size, R^2^ is the coefficient of determination for the regression model, Beta denotes the effect size per allele for the SNP–phenotype association, SE is the standard error of Beta, and EAF represents the effect allele frequency. To ensure analytical accuracy, palindromic SNPs (i.e., those with A/T or C/G alleles) were excluded due to potential inconsistencies in strand orientation across datasets, which could confound the interpretation of exposure–outcome associations (Chen et al. 2024). Ultimately, 15,695 eQTL genes were identified.

#### Selection of palmitoylation genes

Palmitoylation genes were sourced from authoritative review articles (Chen et al. 2024, Li et al. 2023, Chamberlain and Shipston 2015), yielding a total of 31 core regulatory genes. This set includes all 23 members of the ZDHHC acyltransferase family (palmitoylation “writers”) and key depalmitoylating enzymes (palmitoylation “erasers,” such as members of the palmitoyl-protein thioesterase [PPT] family). It comprehensively represents the core regulatory axis of cellular protein palmitoylation, enabling a systematic evaluation of the causal role of the entire palmitoylation pathway in MD.

##### Biological rationale for selecting palmitoylation genes

1. Protein S-palmitoylation is a central, reversible regulatory modification in innate and adaptive immunity, modulating cytokine secretion, immune receptor signaling, and inflammatory pathway activation.
2. Key pathogenic cascades in MD—including IL-1β-driven autoinflammation, Th2 differentiation, and macrophage activation—are all tightly regulated by palmitoylation, indicating a robust biological connection between palmitoylation genes and MD.
3. Targeted pathway analysis enhances statistical power for rare diseases, compensating for the limited number of significant loci typically identified in genome-wide analyses.

#### GWAS summary data for MD

Summary statistics for MD were obtained from the FinnGen Release 12 database (available at: https://storage.googleapis.com/finngen-public-data-r12/summary_stats/release/finngen_R12_H8_MENIERE.gz).

1. Phenotype definition: International Classification of Diseases, 10th Revision (ICD-10) code H81.0 (Meniere’s disease).
2. Sample: 3,680 Meniere’s disease cases and 474,839 matched population controls, all of Finnish ancestry.
3. Quality control: Genotypes were imputed using the SISu v3 imputation panel. Variants with a minor allele frequency (MAF) < 0.001, a Hardy–Weinberg equilibrium P-value < 1 × 10⁻⁶, or an imputation quality score (INFO) < 0.6 were excluded.
4. Rationale for selection: This cohort features standardized phenotype ascertainment and a large sample size, making it well suited for genetic analysis of rare diseases.

#### Immune cell trait data

GWAS summary data for 731 immune cell traits were sourced from the IEU OpenGWAS database (https://gwas.mrcieu.ac.uk/) (Orrù et al. 2020). The immunophenotypes encompass B cells, dendritic cells, mature T cells, monocytes, myeloid cells, TBNK cells, and regulatory T cells. The measured indices included absolute cell counts, relative proportions, median fluorescence intensity (MFI) of surface antigens, and cellular morphological parameters. All samples were derived from populations of European ancestry.

#####  Rationale for focusing exclusively on immune cells

1. Systemic immune dysregulation as a core pathogenic feature of MD: Multiple independent cohorts have demonstrated that MD is not merely a localized inner ear disorder but is accompanied by persistent systemic inflammation. Molecular signatures of peripheral immune cells can reflect the inflammatory status of the inner ear and distinguish distinct immune subtypes (Lopez-Escamez and Cruz-Granados 2025, Frejo et al. 2025, Flook et al. 2024, Jiang et al. 2025, Cruz-Granados et al. 2024). Dysfunction of CD4⁺ T cells and NK cells has been confirmed in affected patients (Flook et al. 2023, Peng et al. 2026).
2. Technical and clinical translational constraints: Human inner ear tissue is extremely difficult to obtain, and no large-scale public eQTL or GWAS datasets for the inner ear currently exist. In contrast, peripheral blood is readily accessible for clinical sampling and can serve as a biomarker for patient stratification.
3. Alignment with the central scientific question: This study aims to determine whether palmitoylation-mediated immune regulatory pathways influence susceptibility to MD. Focusing on immune cells avoids confounding interference from non-immune genes in the inner ear, thereby enhancing the interpretability of the findings.

### Target gene screening

This study was conducted in strict accordance with the STROBE-MR reporting guidelines (Skrivankova et al. 2021). First, from the 22 palmitoylation-related genes identified through intersection with eQTL datasets, we screened for genes significantly associated with MD. Subsequently, summary-data-based Mendelian randomization (SMR) analysis was performed to identify definitive causal target gene(s). The inverse variance weighted (IVW) method was used as the primary Mendelian randomization approach (Burgess et al. 2023), with a statistical threshold of *P* < 0.05 for a causal association between exposure and outcome. Cochran’s Q test was used to assess heterogeneity among the included SNPs; *P* > 0.05 indicated no significant heterogeneity, suggesting consistent effect estimates across the selected SNPs (Greco et al. 2015). The MR-Egger regression intercept was applied to evaluate horizontal pleiotropy, with an intercept *P* > 0.05 indicating an absence of directional pleiotropy—that is, no systematic bias of the SNPs acting as instrumental variables (Bowden et al. 2015). Leave-one-out sensitivity analysis was performed by sequentially removing each individual SNP and rerunning the Mendelian randomization analysis to determine whether any single SNP exerted a disproportionate influence on the results (Yang et al. 2023). SMR analysis integrates GWAS and eQTL data to evaluate polygenic associations between protein expression levels and complex traits, thereby distinguishing whether the effect of a SNP on the phenotype is mediated through protein expression or driven by alternative biological pathways (Qiu et al. 2025).

#### SMR validation using independent eQTL datasets

To mitigate potential bias from reliance on a single eQTL dataset and to verify the robustness of target gene findings, we performed summary-data-based Mendelian randomization (SMR) validation analyses using two independent peripheral blood cis-expression quantitative trait loci (cis-eQTL) datasets as exposure data. Genome-wide association study (GWAS) summary statistics for Meniere’s disease (MD) from the FinnGen database served as the uniform outcome. Analyses were conducted separately for each dataset.

##### eQTL data sources

eQTLGen dataset: Whole-blood cis-eQTL data were obtained from the eQTLGen Consortium (https://www.eqtlgen.org/cis-eqtls.html), comprising 31,684 individuals of European ancestry (Westra et al. 2013). This dataset was used as the discovery set for SMR analysis.

GTEx v8 dataset: Whole-blood cis-eQTL data were derived from the Genotype-Tissue Expression (GTEx) project v8 release (https://gtexportal.org/home/datasets). The whole-blood subgroup included 755 samples of European ancestry. Overall, the GTEx v8 project encompassed 838 donors across 49 human tissues, with eQTL mapping performed using standardized genotype imputation and expression quantification pipelines (GTEx Consortium 2020). This dataset served as an independent validation set for SMR analysis. For both datasets, only cis-eQTL variants located within 1 Mb upstream or downstream of the transcription start site of the target gene were retained.

##### SMR and HEIDI testing procedure

SMR analyses were performed using SMR software (version 1.3.1) following a uniform protocol for both datasets. The SMR method uses cis-eQTL variants as instrumental variables to test for a causal association between gene expression levels and disease risk, thereby distinguishing genuine expression-mediated effects from confounding associations due to linkage disequilibrium (LD) (Zhu et al. 2016).

The heterogeneity in dependent instruments (HEIDI) test was applied to genes showing significant SMR associations to assess whether the observed association was driven by a single causal variant. An association was considered statistically significant at *P*_*SMR*_ < 0.05.*P*_*HEIDI*_> 0.05 was interpreted as evidence against substantial LD confounding, suggesting the association was more likely mediated by gene expression rather than by distinct proximal causal variants. Prior to analysis, we retained variants present in both the eQTL and MD GWAS datasets, excluding ambiguous palindromic variants and those with a minor allele frequency < 0.01. Genes that achieved statistical significance in both datasets and passed the HEIDI test were considered robustly validated target genes.

### Mediation analysis

We performed a two‑step mediation analysis to examine whether immune cell traits mediate the causal effect of palmitoylation‑related genes on MD. First, we used immune cell traits as exposures in a two‑sample MR analysis to identify traits associated with MD. To control the inflated type I error rate in high‑dimensional sequential immune screening, we applied Benjamini–Hochberg (BH) false discovery rate (FDR) correction to the MR P‑values of all 731 immune cell traits (Step 1: immune traits vs. MD). All immune phenotypes were included in the global correction without pre‑filtering nominally significant hits. Only traits meeting FDR < 0.05 were retained for the second‑stage MR and subsequent mediation testing. Since no trait reached the FDR significance threshold, all downstream immune association and mediation analyses were discontinued.

### Software

All Mendelian randomization analyses were performed using the TwoSampleMR and MRPRESSO packages in R software (version 2025.09.2 + 418). Graphical representations were generated with the ggplot2 package. Summary-data-based Mendelian randomization (SMR) analyses were conducted using SMR software, version 1.3.1 (https://cnsgenomics.com/software/smr/#Overview).

## Results

### MR and SMR analyses of palmitoylation-related genes

**Fig. 1:**
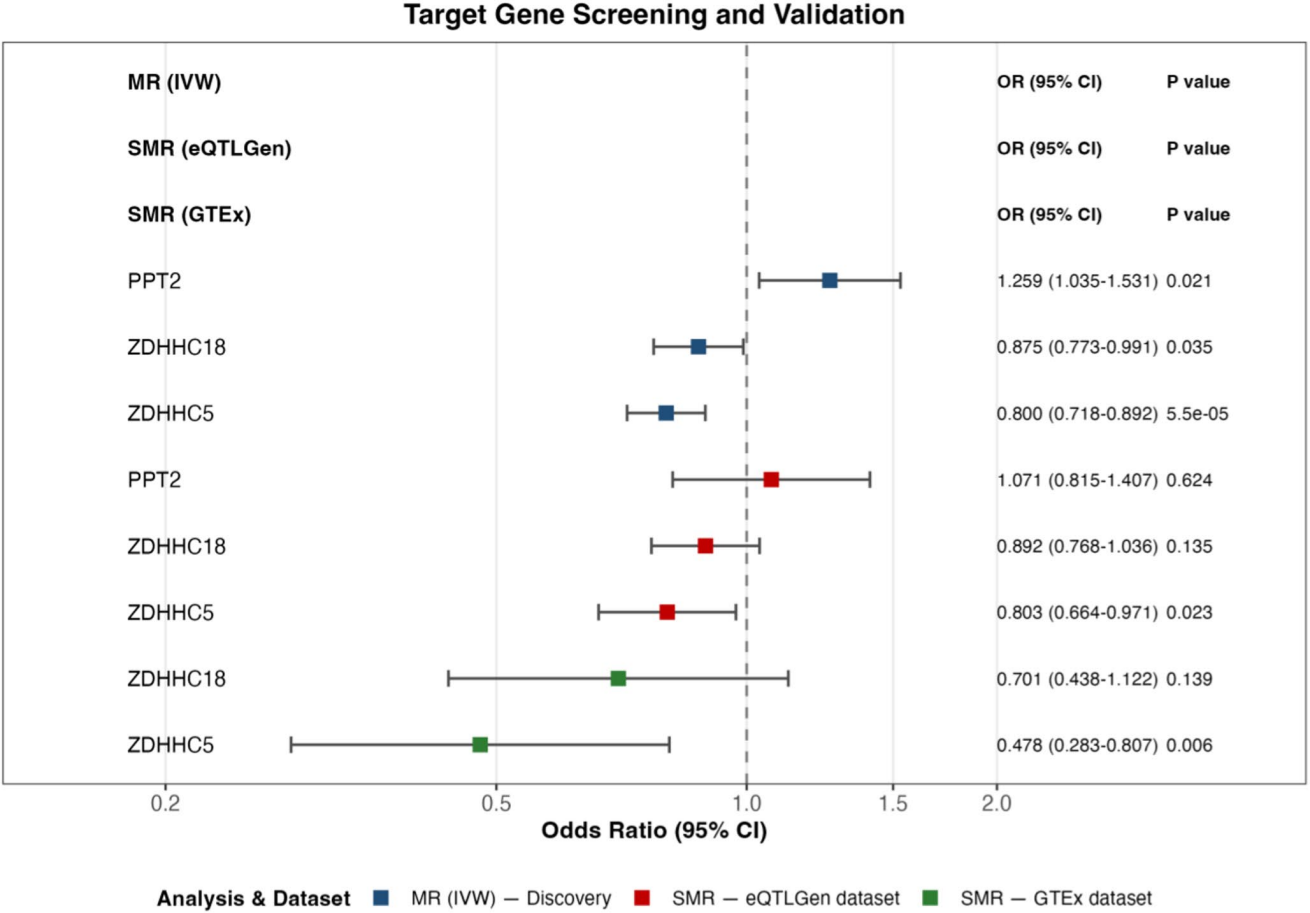
Forest plot of the associations between palmitoylation-related genes and MD

We intersected 15,695 eQTL genes with 31 palmitoylation-related genes, identifying 22 overlapping palmitoylation genes. A two-sample Mendelian randomization (MR) analysis was then performed, revealing significant causal associations with MD for PPT2 (OR = 1.259, 95% CI: 1.035–1.531, *P* = 0.021), ZDHHC18 (OR = 0.875, 95% CI: 0.773–0.991, *P* = 0.035), and ZDHHC5 (OR = 0.80, 95% CI: 0.718–0.892, *P* < 0.001). Subsequently, SMR analysis was conducted, and ZDHHC5 was ultimately identified as the target gene (eQTLGen: OR = 0.803, 95% CI: 0.664–0.971, *P*_*SMR*_ = 0.023; GTEx: OR = 0.478, 95% CI: 0.283–0.807, *P*_*SMR*_ = 0.006) (Fig. 1). HEIDI test *P* > 0.05.

### Sensitivity analysis

**Fig. 2:**
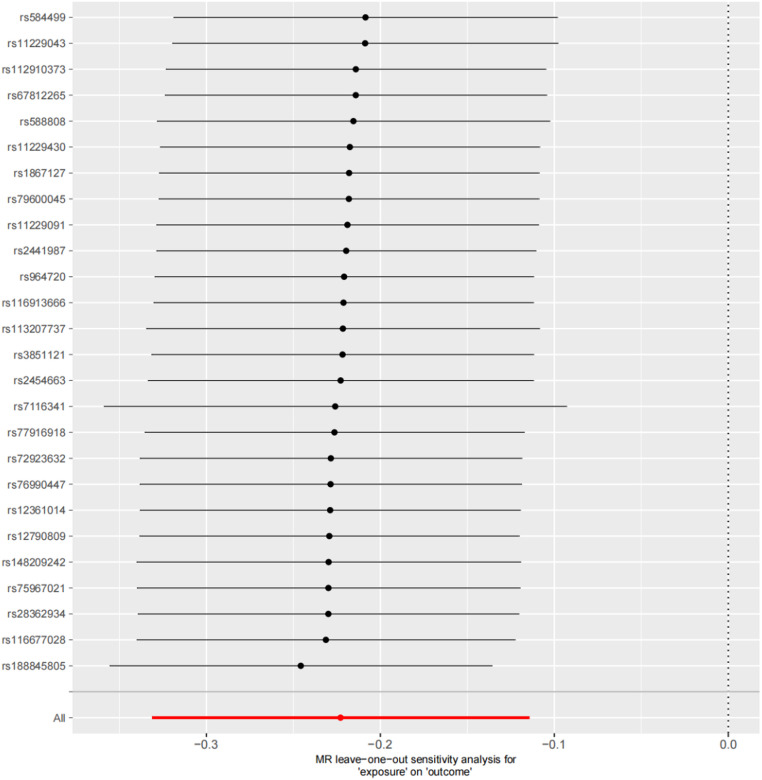
Results of the leave-one-out sensitivity analysis

The results of Cochran’s Q test indicated no significant heterogeneity among the instrumental variables (IVs) (*P* = 0.679). Furthermore, MR-Egger regression intercept analysis revealed no evidence of significant horizontal pleiotropy (*P* = 0.171) (Table 1). Leave-one-out analysis demonstrated that no single SNP disproportionately influenced the overall results (Fig. 2).

**Table 1:** Results of heterogeneity and pleiotropy tests

Cochran’s Q test		MR-Egger	
Ivw (P value)	MR-Egger (P value)	Intercept	P value
0.618	0.679	−0.021	0.171

### Mediation analysis

#### Association between immune cells and MD

We conducted Mendelian randomization analyses using 731 immune cell traits as exposures and MD as the outcome, applying FDR correction; none of the traits met the FDR significance threshold.Therefore, we discontinued subsequent immune association and mediation analyses.

## Discussion

This study integrated large-scale genetic datasets and applied Mendelian randomization to explore, for the first time, the role of protein palmitoylation in Meniere’s disease (MD). It systematically examined the causal relationships between core palmitoylation-regulating genes and MD. Three palmitoylation-related genes were found to be significantly associated with the risk of developing MD. Further validation using SMR identified ZDHHC5 as a core pathogenic target gene, providing initial genetic evidence that links palmitoylation regulatory pathways to MD.

Evidence from the past three years suggests that MD displays significant immune heterogeneity and can be categorized into distinct subtypes, including a Th2-mediated allergic inflammation-driven subtype, an IL-1β-mediated autoinflammatory subtype, and a subtype with concurrent systemic autoimmunity (Lopez-Escamez and Cruz-Granados 2025, Frejo et al. 2025, Flook et al. 2024). Research on T cell populations has further shown that patients with MD can be stratified into three distinct immune phenotypes based on CD4⁺ T cell subset profiles (Jiang et al. 2025). In addition to adaptive T cell abnormalities, innate immune components such as NK cells, macrophages, and the NLRP3 inflammasome have been implicated in the pathogenesis of MD (Flook et al. 2023, Peng et al. 2026, Li et al. 2026, Zhang et al. 2024). For example, elevated circulating IL-1β has been associated with glutamate excitotoxicity and inner ear synaptic damage in MD (Zhang et al. 2024), and the STING signaling pathway in vestibular macrophages represents a key mechanism underlying endolymphatic hydrops (Liu et al. 2026). NKT cells, which share functional characteristics of both NK cells and T cells, may serve as an upstream regulatory node that integrates multiple dysregulated immune pathways in MD.This study was initially designed to test the hypothesis that NKT cells play a mediating role. However, after applying a global false discovery rate (FDR) correction across all 731 immune cell traits, none of the immune phenotypes reached the statistical threshold of FDR < 0.05. Consequently, there is no robust statistical evidence to support NKT cells as a mediating factor, and this proposed immune regulatory pathway remains speculative and unvalidated.

ZDHHC5, a core member of the protein palmitoyltransferase family, has been widely shown to regulate immune cell function and inflammatory signaling pathways (Xiu et al. 2025). Protein palmitoylation broadly modulates immune signaling, including T‑cell receptor signaling and the JAK‑STAT cytokine pathway, which are essential for immune cell development and activation (Collura et al. 2020). Existing functional studies have confirmed that ZDHHC5 promotes NLRP3 inflammasome activation and GSDMD-mediated pyroptosis in inflammatory conditions such as sepsis(Zheng et al. 2023, Cao et al. 2025). Since NLRP3 inflammasome activation and IL-1β secretion are established hallmarks of the autoinflammatory subtype of Meniere’s disease(Frejo et al. 2025, Zhang et al. 2024), we hypothesized that ZDHHC5 may also regulate inner ear inflammatory responses by modulating the inflammasome pathway.

Numerous hypotheses regarding the etiology of Meniere’s disease have long been proposed; however, definitive molecular therapeutic targets remain unestablished. Previous investigations into the immune mechanisms underlying Meniere’s disease were largely limited to correlational observations and lacked causal evidence for validation. Using a genetic approach, the present study is the first to demonstrate that ZDHHC5 may participate in the pathogenic pathway of Meniere’s disease.The pathogenic genes for familial Meniere’s disease—such as OTOG, TECTA, and MYO7A—encode inner ear structural proteins, whose pathways are entirely independent of the immune-related palmitoylation pathway mediated by ZDHHC5 identified in this study. This suggests distinct genetic pathogenic mechanisms between sporadic and hereditary forms of Meniere’s disease.Existing functional research on ZDHHC5 has primarily focused on the nervous system, cardiac function, and tumorigenesis(Xiu et al. 2025, Sun et al. 2025, Liu et al. 2025), with no reports to date describing its role in inner ear disorders. By introducing the study of protein palmitoylation modification into research on Meniere’s disease, this work reveals the potential involvement of ZDHHC5 in its pathogenesis and fills a gap in the understanding of palmitoylation in inner ear diseases.Furthermore, the Mendelian randomization study design effectively mitigates confounding bias and reverse causality inherent in observational studies, thereby improving the robustness of causal inference.

This study has several limitations. First, all eQTL data were derived from peripheral blood rather than inner ear tissue. Given the tissue specificity of gene expression, the expression pattern and function of ZDHHC5 in the inner ear may differ from that observed in peripheral blood. To date, no high-quality, large-scale inner ear eQTL dataset is available, precluding direct assessment of gene expression effects in this tissue. Future studies are needed to validate our findings in inner ear tissues or relevant cellular models, such as cochlear epithelial cells or inner ear resident macrophages.Second, the Mendelian randomization approach used in this study captures only common genetic variants, which may exert pleiotropic effects across multiple genes and pathways. Although we applied MR-Egger regression and Cochran’s Q test to assess pleiotropy and heterogeneity, residual pleiotropy cannot be entirely excluded.Third, the summary-level data for Meniere’s disease were derived exclusively from a Finnish population (FinnGen R12), and the immune cell data were also obtained from individuals of European ancestry. Although both datasets are from European populations, bias due to population stratification cannot be ruled out. The causal associations identified here require replication in independent Meniere’s disease cohorts, particularly in East Asian populations, to assess their generalizability.Fourth, the present MD GWAS pooled all patients into a single phenotype without stratifying the three major immune subtypes—autoinflammatory, quiescent, and Th2-skewed. Should the genetic effect of ZDHHC5 be specific to only one subtype, a pooled analysis across the entire cohort would dilute the true effect size and bias subsequent mechanistic interpretations. Future studies could leverage the single cell transcriptome dataset published by Cruz‑Granados et al. (2024) to compare ZDHHC5 expression levels across subtypes and clarify its subtype‑specific functions.Fifth, this study included a total of 731 peripheral immune cell phenotypes for two‑step mediation screening. No statistically significant immune targets survived global FDR correction across all traits. Traits with only nominal significance (*P* < 0.05) carry a high risk of false positives; therefore, subsequent mediation analyses were not conducted. Consequently, the specific immune cell populations that may mediate the association between ZDHHC5 and MD could not be identified, leaving the proposed immune‑related mechanisms without statistical support.

## Conclusion

In summary, two-sample MR and two-cohort SMR analyses collectively confirm a statistically significant genetic association between peripheral blood ZDHHC5 gene expression and Meniere’s disease (MD). We attempted to identify immune cell phenotypes that might mediate this association; however, no statistically significant immune targets survived global FDR correction across all 731 immune traits. Consequently, the potential involvement of the NKT cell-related immune regulatory pathway remains an unvalidated hypothesis. This study provides only genetic correlative evidence, and all mechanistic interpretations are limited by several factors, including the lack of functional validation, potential confounding from pooling all MD immune subtypes, and the risk of false positives inherent in high-throughput screening. Further research is needed to: 1. Perform genetic analyses stratified by immune subtypes and validate subtype-specific differential expression of ZDHHC5 using single-cell datasets; 2. Replicate the genetic association between ZDHHC5 and MD in independent population cohorts; 3. Conduct cellular and animal functional studies to clarify the role of ZDHHC5-mediated palmitoylation in immune regulation and inner ear pathology, thereby elucidating definitive pathological mechanisms and potential therapeutic interventions.

## Data Availability

All data supporting the findings of this study are available within the paper and its Supplementary Information.
